# Early Versus Late Diagnosis of Youth‐Onset Type 2 Diabetes

**DOI:** 10.1002/edm2.70116

**Published:** 2025-10-03

**Authors:** Nisha Krishnan, Nancy A. Crimmins, Debi Swertfeger, Lisa Schaaf, Amy S. Shah

**Affiliations:** ^1^ Division of Endocrinology Cincinnati Children's Hospital Medical Center Cincinnati Ohio USA; ^2^ Department of Pediatrics Cincinnati Children's Hospital Medical Center Cincinnati Ohio USA

**Keywords:** metabolic, paediatrics, risk factors, type 2 diabetes

## Abstract

**Objective:**

To determine if earlier age of diabetes onset (< 15 years of age) is associated with a worse metabolic phenotype compared to diabetes diagnosed at a later age (≥ 15 years of age).

**Methods:**

Retrospective cross‐sectional study of our clinical cohort was performed at a tertiary clinic between 2018 and 2023. Diabetes presentation (diabetic ketoacidosis, C‐peptide levels, ketonuria), metabolic phenotype (body mass index (BMI) *z*‐score, hypertension, dyslipidemia, elevated liver enzymes, and haemoglobin A1c) were compared between youth diagnosed with type 2 diabetes who were < 15 years of age and those ≥ 15 years of age. A *p* value of < 0.05 was considered significant.

**Results:**

We studied *n* = 336 youth. Mean age was 14.5 years at type 2 diabetes diagnosis, *n* = 198 were < 15 years and *n* = 138 were ≥ 15 years old. Youth diagnosed at < 15 years versus ≥ 15 years old had a lower systolic and diastolic blood pressure (121.0 ± 12.0 vs. 125.0 ± 12.3 mmHg, *p* = 0.004 and 72.0 ± 9.8 vs. 74.9 ± 11.1 mmHg, *p* = 0.013 respectively), and higher HDL cholesterol (38.3 ± 11.7 vs. 35.7 ± 8.7 mg/dL, *p* = 0.049). There were no differences in the frequency of diabetic ketoacidosis, urine ketones at presentation, C‐peptide concentrations, haemoglobin A1c, liver enzymes, total or LDL cholesterol, or BMI *z*‐scores by age group.

**Conclusions:**

A worse metabolic profile was not observed in youth diagnosed at a younger age. In fact, youth who were at an older age at diabetes diagnosis tended to have higher blood pressure and lower HDL‐C. Establishing the risk factors for why some youth develop type 2 diabetes at earlier ages is needed.

## Graphical abstract

A retrospective cross‐sectional study of youth‐onset type 2 diabetes (T2D) presentation stratified by age at diagnosis (< 15 years vs. ≥ 15 years). Younger age at diabetes onset within this cohort of youth with T2D was not associated with greater frequency of metabolic abnormalities, lower C‐peptide concentrations, or a more severe diabetes presentation.

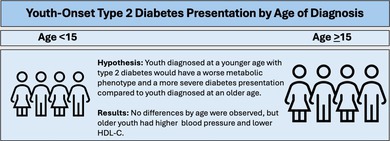

## 1 Introduction

The incidence and prevalence of type 2 diabetes (T2D) with onset in youth continue to increase in the United States and around the world [1, 2, 3]. Similar to adult‐onset T2D, youth‐onset T2D results from a combination of insulin resistance and impaired insulin secretion leading to the development of hyperglycemia. However, unlike adult‐onset T2D, youth with T2D have greater insulin resistance, faster rates of pancreatic beta cell decline, and are at risk of developing nephropathy, retinopathy, and neuropathy within 5–10 years of diabetes onset [4, 5, 6, 7, 8, 9]. Furthermore, at T2D diagnosis, 11%–20% of youth have diabetic ketoacidosis and 10%–13% have hypertension and dyslipidemia, demonstrating that co‐morbidities and complications are evident at diagnosis [10, 11, 12, 13].

Diagnosis of type 2 diabetes before onset of puberty/age < 10 years has been observed [14, 15, 16, 17, 18]. Though rare, these youth are more likely to be female, from ethnic minority race groups or indigenous populations, and have a higher body mass index. Even within adult cohorts, younger age at diabetes onset is associated with greater hyperglycemia and obesity, and higher rates of complications at diagnosis [19]. Thus, age at diabetes onset may be an important marker of more aggressive diabetes.

In this study, we sought to examine whether age at diabetes diagnosis within a cohort of patients with youth‐onset type 2 diabetes was associated with a more severe diabetes phenotype at presentation. Our hypothesis was that youth diagnosed with T2D at an earlier age (< 15 years of age) would have higher body mass index *z*‐scores, a greater number of metabolic abnormalities at diagnosis, lower C‐peptide levels and perhaps a more severe presentation of diabetes compared to youth with T2D diagnosed in mid to late adolescence (≥ 15 years of age). Age 15 years was chosen as the cutoff for categorization as this likely represents the age during (< 15 years) and following peak insulin resistance (≥ 15 years) [20, 21], If true, this would suggest an even more aggressive disease process in youth diagnosed with type 2 diabetes in early adolescence and perhaps a need for additional interventions to prevent future diabetes‐related complications.

## 2 Methods/Design

A retrospective cross‐sectional analysis was conducted in youth (defined as less than 19 years of age) diagnosed with new onset T2D at Cincinnati Children's Hospital Medical Center (CCHMC) between January 2018 and April 2023, starting when our type 2 diabetes multidisciplinary program began [22]. The diagnosis of diabetes was defined according to the American Diabetes Association (ADA) criteria [23]. All youth included in the analysis had a negative islet cell antibody panel (glutamic acid decarboxylase‐65 (GAD‐65), islet antigen‐2 (IA‐2), zinc transporter 8 (ZnT8) and insulin antibody (IAA)) and a clinical phenotype consistent with type 2 diabetes (elevated BMI, signs of insulin resistance) confirmed by the research team. For this paper, younger youth will be used interchangeably with the cohort < 15 years of age at diagnosis, and older youth will be used synonymously with the cohort ≥ 15 years of age at diagnosis.

Demographic data including age, race/ethnicity, and biological sex were abstracted from the electronic medical record. Clinical data including weight, calculated BMI *z*‐score, systolic blood pressure (SBP), diastolic blood pressure (DBP), haemoglobin A1c (HbA1c), C‐peptide levels, the presence of urine ketones (trace, small, moderate or large ketones) and diabetic ketoacidosis (DKA) were also abstracted from the electronic medical record at the time of diagnosis. Laboratory values including total cholesterol (total‐C), high density lipoprotein cholesterol (HDL‐C), low density lipoprotein cholesterol (LDL‐C), triglycerides, alanine aminotransferase (ALT), aspartate aminotransferase (AST) and albumin‐to‐creatinine ratio (A/C ratio) were also collected. If lipids, liver, and urine tests were not performed on the date of diagnosis, results within 6 months after the diagnosis date were included. For *n* = 40 youth, lipids were obtained in the 6 months prior to their diabetes diagnosis date. If multiple lab values were available during this time frame, we only used the labs that were drawn closest to the diagnosis date.

Metabolic abnormalities were defined as having abnormal cardio‐metabolic labs including SBP, total‐C, HDL‐C, LDL‐C, triglycerides, ALT, AST, and/or A/C ratio. Clinical labs were defined as abnormal if they exceeded the following thresholds: SBP ≥ 120 mmHg, total‐C ≥ 200 mg/dL, HDL‐C ≤ 40 mg/dL, LDL‐C ≥ 130 mg/dL, triglycerides ≥ 150 mg/dL, ALT or AST ≥ 40 unit/L, or A/C ratio ≥ 30 mg/g using cutoffs of significance from the ADA [24].

## 3 Statistics

Data are reported for the overall cohort as mean ± standard deviation or *n* (%) and then stratified by age. Comparisons of means by age were evaluated using unpaired *t*‐tests, while differences in proportions (race/ethnicity, sex) by age were evaluated by Mann–Whitney *U* test. Pearson correlation coefficients between age and the following variables (total‐C, HDL‐C, LDL‐C, triglycerides, SBP, BMI *z*‐scores, ALT and AST) were used to determine if age was related to a worse metabolic profile. In sub‐analyses, we also examined results among non‐Hispanic white, non‐Hispanic Black and among females (our largest subgroups). Finally, results were also examined using a lower age cutoff of < 13 years versus ≥ 13 years to assess whether age selection influenced our results. For all analyses, values of *p* < 0.05 indicated statistical significance.

## 4 Results

The cohort included 336 youth with new onset T2D who were a mean age of 14.5 years at T2D onset, *n* = 198 < 15 years and *n* = 138 ≥ 15 years old. The age distribution of the cohort is shown in Figure S1. The clinic cohort was majority female (63.4%). 46.4% (*n* = 156) of the population self‐identified as non‐Hispanic black, 35.4% (*n* = 119) self‐identified as non‐Hispanic white, 9.2% (*n* = 31) self‐identified as mixed race/ethnicity, other (including Asian, Hawaiian, Middle Eastern, etc.) or unknown, and 8.9% (*n* = 30) self‐identified as Hispanic white (Table 1). When stratified by age, there was no difference between race/ethnicity distribution and age at diagnosis. The proportion of male and female youth with T2D did not differ by age group.

**TABLE 1:** Demographic characteristics of youth at diagnosis, overall and stratified by age.

	All patients (*n* = 336)	Youth diagnosed age < 15 (*n* = 198)	Youth diagnosed age ≥ 15 (*n* = 138)	*p*
Age (years)	14.5 ± 2.2	13.0 ± 1.5	16.7 ± 1.0	
Race/Ethnicity				0.49
Hispanic White	30 (8.9%)	18 (9.1%)	12 (8.7%)	
Non‐Hispanic Black	156 (46.4%)	101 (51.0%)	55 (39.9%)	
Non‐Hispanic White	119 (35.4%)	57 (28.8%)	62 (44.9%)	
Other^a^	31 (9.2%)	22 (11.1%)	9 (6.5%)	
Biological Sex				0.909
Female	213 (63.4%)	126 (63.6%)	87 (63.0%)	
Male	123 (36.6%)	72 (36.4%)	51 (37.0%)	

*Note:* Data are Mean ± SD or *n* (%).

^a^ Other includes self‐reported mixed race/ethnicity *n* = 16 (*n* = 13 were non‐Hispanic Black and White; *n* = 1 non‐Hispanic Black and Asian, *n* = 1 non‐Hispanic Black, White and Native American, and *n* = 1 non‐Hispanic Black, White, and Hawaiian *n* = 1), Asian *n* = 5, Hawaiian *n* = 3, Unknown *n* = 2, Hispanic Other *n* = 2, Hispanic Black *n* = 1, Hispanic Native American *n* = 1, Middle Eastern *n* = 1.

**FIGURE 1:**
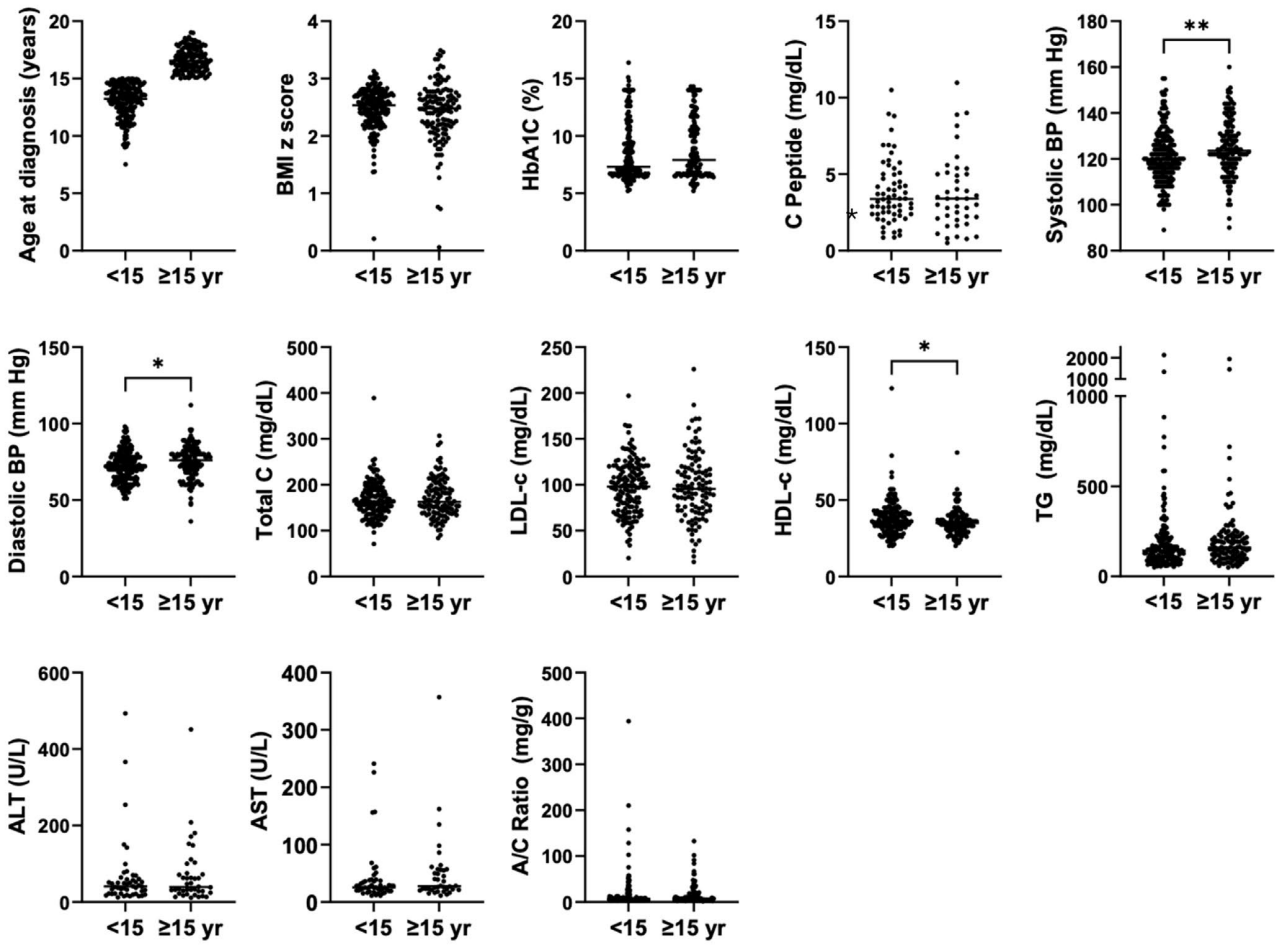
Distribution of clinical lab values separated by age of type 2 diabetes diagnosis. Data shown represents all youth whose data was available in the electronic medical record. BMI *z*‐score data was available for *n* = 194 youth < 15 years and *n* = 132 youth > 15 years. Lipid data was available for *n* = 152 youth < 15 years and *n* = 114 youth ≥ 15 years. ALT and AST data were available for *n* = 49 youth < 15 years and *n* = 39 age ≥ 15 years. C‐Peptide data was available for *n* = 62 youth < 15 years and *n* = 42 youth ≥ 15 years. Statistically significant difference was denoted as follows: *p < 0.05, **p < 0.01.

Metabolic characteristics in the overall cohort were examined and then further stratified by age group (Table 2). Youth diagnosed with T2D at < 15 years had a lower systolic and diastolic blood pressure (121.0 ± 12.0 vs. 125.0 ± 12.3 mmHg, *p* = 0.004 and 72.0 ± 9.8 vs. 74.9 ± 11.1 mmHg, *p* = 0.013 respectively), and higher HDL‐C (38.3 ± 11.7 vs. 35.7 ± 8.7 mg/dL, *p* = 0.049) as compared to those diagnosed ≥ 15 years old. BMI *z*‐score, HbA1C levels, liver enzymes (ALT/AST), total‐C, LDL‐C, triglycerides and A/C ratio did not differ by age of diabetes onset. There was also no statistically significant difference in frequency of DKA at diagnosis, presence of urine ketones at presentation, or C‐peptide levels in younger versus older youth. Scatter plots of individual data points are shown in Figure 1.

**TABLE 2:** Metabolic characteristics of youth at diagnosis, overall and stratified by age.

	All youth (*n* = 336)	Youth diagnosed at age < 15 (*n* = 198)	Youth diagnosed at age ≥ 15 (*n* = 138)	*p* between groups
Weight (kg)^a^	109.2 ± 1.5	101.9 ± 26.7	119.8 ± 34.9	< 0.001
BMI z‐score^b^	2.5 ± 0.4	2.5 ± 0.4	2.4 ± 0.5	0.29
Haemoglobin A1c (%, mmol/mol)	8.7 ± 2.6, 72 ± 5	8.6 ± 2.6, 70 ± 5	8.9 ± 2.6, 74 ± 5	0.39
Systolic BP (mmHg)	122.6 ± 12.3	121.0 ± 12.0	125.0 ± 12.3	0.004
Diastolic BP (mmHg)	73.2 ± 10.4	72.0 ± 9.8	74.9 ± 11.1	0.013
Total‐C (mg/dL)^c^	169.1 (40.3)	168.1 (38.5)	170.4 ± 42.7	0.66
HDL‐C (mg/dL)^c^	37.2 (10.6)	38.3 (11.7)	35.7 ± 8.7	0.049
LDL‐C (mg/dL)^c^	97.5 ± 32.3	97.3 ± 29.2	97.8 ± 36.1	0.897
Triglycerides (mg/dL)^c^	202.7 ± 228.4	197.1 ± 226.7	210.2 ± 231.3	0.65
ALT (unit/L)^d^	67.0 ± 84.2	65.1 ± 88.4	69.3 ± 79.8	0.82
AST (unit/L)^d^	44.9 ± 54.1	41.3 ± 48.7	49.7 ± 60.8	0.47
A/C ratio (mg/g)	19.6 ± 38.0	21.3 ± 47.0	17.5 ± 23.5	0.48
C‐Peptide Levels (mg/dL)^e^	3.8 ± 2.2	3.8 ± 2.1	3.8 ± 2.4	0.93
DKA present at T2D diagnosis	26 (7.7%)	14 (7.1%)	12 (8.7%)	0.68
Ketones Present^f^	80 (46.5%)	44 (45.8%)	36 (47.4%)	0.88

*Note:* HbA1C conversions as follows: 5% = 31 mmol/mol, 10% = 86 mmol/mol, 15% = 140 mmol/mol, 20% = 195 mmol/mol.

^a^ Weight data was available for *n* = 194 youth < 15 years and *n* = 134 youth > 15 years.

^b^ BMI *z*‐score data was available for *n* = 194 youth< 15 years and *n* = 132 youth ≥ 15 years.

^c^ Lipid data was available for *n* = 152 youth < 15 years and *n* = 114 youth ≥ 15 years.

^d^ ALT and AST data were available for *n* = 49 youth < 15 years and *n* = 39 youth ≥ 15 years.

^e^ C‐Peptide data was available for *n* = 62 youth < 15 years and *n* = 42 youth ≥ 15 years.

^f^ Ketones data was available for *n* = 96 youth < 15 years and *n* = 76 youth ≥ 15 years.

**FIGURE 2:**
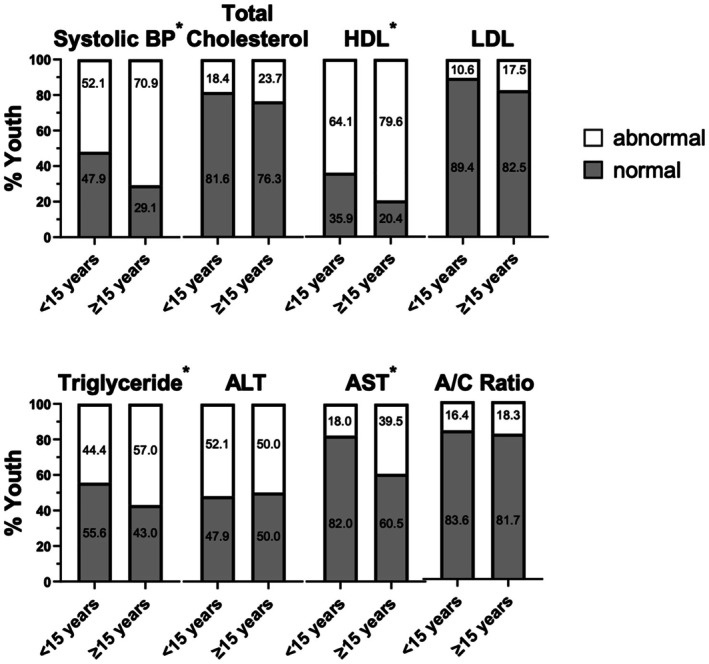
Frequency of abnormal metabolic markers. Laboratory values in youth < 15 years of age. Data are shown as frequency normal and abnormal. Abnormal was defined as Systolic BP ≥ 120 mmHg, Total Cholesterol ≥ 200 mg/dL, HDL ≤ 40 mg/dL, LDL ≥ 130 mg/dL, Triglycerides ≥ 150 mmol/L, ALT ≥ 40 unit/L, AST ≥ 40 unit/L and A/C Ratio ≥ 30 mg/g. *Statistically significant difference determined by Fischer exact tests: Age ≥ 15 years with higher percentage of abnormal SBP (*p* = 0.0009), HDL‐C (*p* = 0.006), triglycerides (*p* = 0.048) and AST (*p* = 0.031).

The frequencies of abnormal metabolic markers at diagnosis, using clinical cutoffs, were then analysed (Figure 2). We hypothesized that younger children would have a higher frequency of abnormal metabolic lab values; however, youth diagnosed at age ≥ 15 years had a higher percentage of abnormal SBP (*p* = 0.0009), HDL‐C (*p* = 0.006), triglycerides (*p* = 0.048), and AST (*p* = 0.031). There were no differences in the frequency of abnormal levels of total‐C, LDL‐C, ALT or A/C ratio by age group.

Pearson correlation coefficients in this study showed that age of diabetes onset was associated with worse metabolic parameters, including blood pressure and BMI *z*‐scores, at diagnosis. Older age was positively and significantly associated with higher systolic and diastolic BP (both *r* = +0.17, *p* = 0.002) and higher BMI *z*‐score (*r* = −0.11, *p* = 0.047). Age was not associated with A/C ratio, haemoglobin A1c, liver function tests (ALT and AST) or other lipid parameters (total‐C, HDL‐C, LDL‐C, and triglycerides).

Sub‐analyses were conducted among females (*n* = 213), and then among non‐Hispanic Black (*n* = 156) and non‐Hispanic white youth (*n* = 119) < 15 years versus ≥ 15 years of age to determine if metabolic characteristics were different within these sub‐groups. Among females, youth with T2D diagnosed < 15 years had higher mean BMI z‐scores (2.5 ± 0.3 vs. 2.3 ± 0.5, *p* < 0.01) and lower SBP and DBP (119.7 ± 11.6 vs. 124.4 ± 12.2 mmHg, *p* < 0.01 and 71.5 ± 9.7 vs. 75.8 ± 11.0 mmHg, *p* < 0.01, respectively). All other metabolic characteristics were similar. Among youth who identified as non‐Hispanic Black, the only observed difference was youth diagnosed with T2D at < 15 years had lower SBP (120.2 ± 11.3 vs. 124.3 ± 13.23 mmHg, *p* < 0.05). Among non‐Hispanic white youth, youth diagnosed at < 15 years had lower DBP (71.2 ± 8.7 vs. 75.8 ± 9.5 mmHg, *p* = 0.008) and higher HDL‐C (37.5 ± 9.0 vs. 33.7 ± 8.0 mg/dL, *p* = 0.03). In general, these sub‐analyses were consistent with findings in the overall cohort.

Finally, analyses were evaluated using a younger age of diagnosis of < 13 years versus ≥ 13 years of age. Youth < 13 years at T2D diagnosis had lower total cholesterol (158.9 ± 28.8 vs. 172.3 ± 42.9 mg/dL, *p* = 0.02) and higher A/C ratio (31.0 ± 68.7 vs. 16.2 ± 21.5 mg/g, *p* = 0.02) compared to youth diagnosed at ≥ 13 years of age.

## 5 Discussion

In this study, we aimed to determine whether youth diagnosed with type 2 diabetes at a younger age had more metabolic abnormalities, lower C‐peptide levels, and/or a more severe presentation of diabetes. The goal was to identify clinical or metabolic characteristics in younger youth that, if present, may suggest why T2D onset may occur during versus after the time of peak insulin resistance.

We found that youth diagnosed with diabetes at age < 15 years were more likely to be non‐Hispanic Black. Similar data have been published in the SEARCH for Diabetes in Youth study where the mean age of diabetes onset was 15 years except for non‐Hispanic Black females where age at diagnosis was at age 13 years [1]. Additionally, when diabetes is diagnosed before puberty onset, it is more likely to be seen in minority ethnic youth [14, 15, 16, 17, 18]. Known risk factors for youth‐onset T2D, as outlined in the ADA and International Society of Paediatric and Adolescent Diabetes, include having a positive family history of type 2 diabetes, physical signs of insulin resistance such as acanthosis nigricans, a maternal history of gestational diabetes, and belonging to a minority ethnic race group such as Black, Hispanic/Latino, Native American/Alaska Native, Asian American or Pacific Islander [23, 25]. In this single center study, we observed that younger youth tended to be non‐Hispanic Black, but when the diagnosis of diabetes was at age ≥ 15 years, the race/ethnic distribution tended to be non‐Hispanic white. While these findings should be confirmed in additional studies across multiple geographic locations and/or centers, these data emphasize that perhaps race/ethnic risk stratification for diabetes screening may only apply to younger youth.

Though we found a high frequency of co‐morbidities in our clinical cohort, including lipid abnormalities, elevated blood pressure, and elevated liver enzymes regardless of age, none of these labs were worse in younger youth. In fact, it was older age of onset that was associated with higher SBP and lower HDL‐C, and a greater proportion of abnormalities in SBP, HDL‐C, triglycerides and AST. Higher SBP may reflect the strong correlation between age and blood pressure [26, 27], and worse lipids and AST may be linked to the high BMI of the cohort [28, 29, 30]. It is unclear why these risk factors are associated with older age of diabetes onset, but they may be physiologic, as higher rates of hypertension and dyslipidemia are observed with increasing age [31].

We found no evidence that youth with a younger age of T2D onset had lower C‐peptide concentrations, or a higher frequency of DKA or ketonuria at diagnosis. Since C‐peptide levels were similar between the groups, this suggests that differences in beta cell function may not explain the younger age at presentation [17]. However, it is also possible that differences in beta cell function might become evident if assessed under provoked conditions, such as during an oral glucose tolerance test or unmasked during peak pubertal insulin resistance as others have suggested [32]. When using a lower age cutoff of 13 years, we found higher mean albuminuria levels in youth < 13 years of age at diagnosis. Whether albuminuria is related to younger age at diabetes onset needs to be confirmed in other studies.

There are limitations to this study. First, we used age to represent peak and post‐peak insulin resistance, not Tanner staging data. While age and puberty strongly correlate, age is not a perfect surrogate. For that reason, we also evaluated results using a cutoff at a younger age of diagnosis, but results remained largely the same. Second, we lacked descriptive family history details, which could be a strong component contributing to age at T2D diagnosis, and we lacked information on lifestyle factors (dietary habits and exercise frequency). Finally, we lacked complete laboratory values on all participants and data such as BMI prior to the date of diagnosis, which could have assessed the trajectory of weight gain. However, the strengths of this study include the initial evaluation of the clinical differences by age of T2D diagnosis, which has not yet been explored.

In conclusion, younger age at diabetes onset within a cohort of youth with T2D was not associated with greater frequency of metabolic abnormalities, lower C‐peptide concentrations, or a more severe diabetes presentation. These findings reinforce that although the risk factors associated with youth‐onset T2D are known, the exact aetiology as to why some youth present at a younger age is undefined and remains an area for future investigation.

## Author Contributions

Nisha Krishnan led data retrieval. Nisha Krishnan, Nancy A. Crimmins, Amy S. Shah contributed to study conceptualization, analysis, writing, and editing. Lisa Schaaf contributed to writing and editing the manuscript. All authors read and approved the final manuscript.

## Ethics Statement

The Institutional Review Board at Cincinnati Children's Hospital Medical Center (IRB ID: 2023–0208) reviewed this study and determined an “Exempt” status.

## Consent

The authors have nothing to report.

## Conflicts of Interest

The authors declare no conflicts of interest.

## Supporting information


**Figure S1:** Age Distribution at Diabetes Diagnosis.

## Data Availability

The datasets generated and/or analysed during the current study are not publicly available due to institutional requirements but are available from the corresponding author on reasonable request.
