# Supplementary figures and images for: Early Versus Late Diagnosis of Youth‐Onset Type 2 Diabetes
Source: Endocrinol Diabetes Metab. 2025 Oct 3;8(6):e70116. doi: 10.1002/edm2.70116 (PMC12492476; doi:10.1002/edm2.70116)

Supplemental Figure 1. Age Distribution at Diabetes Diagnosis


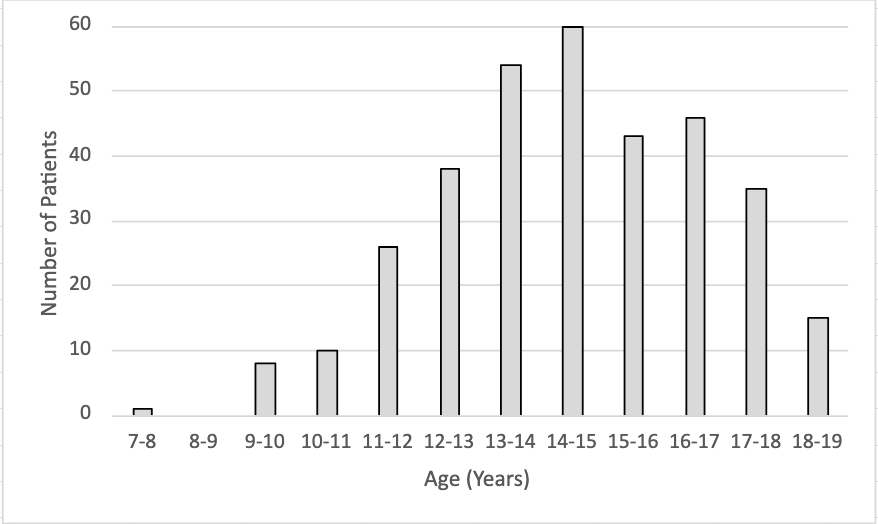

Supplement: Supplementary file 1 — Figure S1: Age Distribution at Diabetes Diagnosis. [file EDM2-8-e70116-s001.docx]
